# Long COVID and the Challenge of Long-Term Employment: An Ecological, Sequential Explanatory Mixed-Methods Approach

**DOI:** 10.1007/s10926-026-10376-w

**Published:** 2026-03-12

**Authors:** Han Su, Briana Keller, Valerie Danesh, Joanne McPeake, Leanne M. Boehm, Tammy L. Eaton, Matthew F. Mart, Mayur B. Patel, E. Wesley Ely

**Affiliations:** 1 School of Nursing, Vanderbilt University, 461 21 St Ave. S, Nashville, TN 37240, USA; 2 College of Education, University of Alabama, Tuscaloosa, AL, USA; 3 Center for Applied Health Research, Baylor Scott and White Health, Dallas, TX, USA; 4 Department of Medicine, Baylor College of Medicine, Houston, TX, USA; 5 The Healthcare Improvement Studies Institute, University of Cambridge, Cambridge, UK; 6 Critical Illness, Brain Dysfunction, and Survivorship (CIBS) Center, Center for Health Services Research, Institute for Population and Public Health, Vanderbilt University Medical Center, Nashville, TN, USA; 7 School of Medicine, Vanderbilt University, Nashville, TN, USA; 8 VA Center for Clinical Management Research, VA Ann Arbor Healthcare System, Ann Arbor, MI, USA; 9 Department of Internal Medicine, Division of Hospital Medicine, Michigan Medicine, Ann Arbor, MI, USA; 10 Institute for Healthcare Policy and Innovation, University of Michigan, Ann Arbor, MI, USA; 11 Division of Allergy, Pulmonary, and Critical Care Medicine, Department of Medicine, Vanderbilt University Medical Center, Nashville, TN, USA; 12 Geriatric Research, Education, and Clinical Center (GRECC), Tennessee Valley Veterans Affairs Healthcare System, Nashville, TN, USA; 13 Division of Acute Care Surgery, Department of Surgery, Section of Surgical Sciences, Vanderbilt University Medical Center, Nashville, TN, USA

**Keywords:** Disabilities, Job accommodation, Vocational rehabilitation, Return to work

## Abstract

**Purpose:**

To identify and contextualize the determinants of long-term employment, health, and financial outcomes among individuals affected by Long COVID.

**Methods:**

Sequential explanatory mixed-methods study design guided by the social–ecological model. Adults with Long COVID who were employed before infection and returned to work during the three-year follow-up were recruited through in-person and virtual outpatient venues: ResearchMatch, a Long COVID clinic, and a peer support group affiliated with a medical center. Participants completed validated surveys assessing factors influencing sustained employment. Stratified semi-structured interviews were then conducted to explore how these factors shaped sustained employment. Quantitative data were analyzed using descriptive and inferential statistical methods, while qualitative data were analyzed through content analysis.

**Results:**

Among 79 participants who returned to work, 58% (*n* = 46) remained employed after a mean follow-up of 1,077 days. Those still employed reported reduced capacity and persistent uncertainty. Those no longer employed experienced worse physical health (*p* < 0.002), greater comorbidity burden (*p* = 0.01), more environmental barriers (*p* = 0.02), and increased financial hardship (*p* = 0.03). Qualitative analyses identified nonlinear return-to-work trajectories shaped by fluctuating and often invisible symptoms, alongside multilevel themes influencing employment sustainability, including misalignment between functional capacity and job demands, challenges obtaining workplace accommodations, stigma, limited policies, and labor market barriers.

**Conclusions:**

Employment sustainability among individuals with Long COVID is shaped by complex, multilevel barriers, with job loss further worsening health and financial hardship. Investment in comprehensive Long COVID care, including multidisciplinary clinical services, vocational rehabilitation, clinician education, public awareness initiatives, employer training, and policy reform, is critical to support long-term recovery and employment sustainability.

## Introduction

Nearly one in seven working-age Americans is affected by Long COVID [1]. The Centers for Disease Control and Prevention define Long COVID as a chronic condition occurring after acute SARS-CoV-2 infection and lasting ≥ 3 months [2]. This condition commonly involves cognitive impairment similar to dementia and multisystem dysfunction (e.g., autonomic, immune, cardiovascular, pulmonary), with symptoms that often fluctuate over time [3–5]. Such manifestations can significantly impair occupational functioning, increasing the risk of job loss [6–8]. Disruptions in employment may jeopardize financial security and limit access to healthcare, compounding existing health vulnerabilities [9, 10].

Global data indicate that approximately 60.9% of individuals with Long COVID resume employment ≥ 12 weeks post-infection [8, 11, 12]. However, qualitative studies from the United Kingdom and a recent rapid review describe the return-to-work process as challenging, shaped by fluctuating symptoms and uneven access to occupational health and workplace support [13–15]. As the Long COVID population evolves, these return-to-work challenges may be further amplified by prolonged and complex symptom trajectories. Recent occupational health guidance from the Society of Occupational Medicine, the World Health Organization Living Guideline, and the American College of Occupational and Environmental Medicine emphasizes that return to work should be understood as a sustained, adaptive process requiring early, work-focused clinical intervention, coordinated occupational health support, and prevention of reinfection (including reinfection prevention and timely management of emerging health problems) [16–18]. Despite these recommendations, sustained employment over time has received limited empirical attention in the Long COVID literature, which has largely focused on initial return to work.

Evidence from other clinical populations, such as those with work-related injuries, cancer, brain injury, and chronic kidney disease, indicates that the inability to sustain employment following initial workforce reentry is both prevalent and multifactorial [19–22]. Guided by the social–ecological model [23], employment sustainability is shaped by interacting influences across individual, interpersonal, community, and societal levels. At the individual level, factors such as demographic characteristics, functional capacity, symptom burden, and job demands are key determinants of continued employment [19, 23]. Interpersonal influences, including the availability and quality of social support, further shape long-term occupational outcomes [23, 24]. Community-level factors—such as workplace environments, organizational processes, and access to accommodations—may shape how health-related limitations are experienced at work, while broader societal determinants, including policy context, labor market conditions, and cultural norms, further influence employment trajectories among affected populations [19, 23, 24].

Taken together, given the chronic and often disabling nature of Long COVID, the absence of effective treatments [2], and the well-documented health consequences of unemployment [9, 10], understanding sustained employment following return to work—shaped by interacting influences across individual, interpersonal, community, organizational, and societal levels—is critical to informing interventions that promote long-term workforce participation. Accordingly, this study aimed to: (1) characterize patterns of sustained employment; (2) examine the multilevel factors associated with maintaining employment; and (3) investigate the health and social outcomes associated with failure to sustain employment among individuals with Long COVID.

## Methods

### Study Design and Participants

A sequential explanatory mixed-methods study design was employed. We first used validated questionnaires to identify factors associated with long-term employment sustainability among participants who self-reported having Long COVID. We then conducted semi-structured interviews with a subset of participants who had completed the questionnaires to gain deeper insight into how the multilevel factors identified in the quantitative data influenced sustained employment among participants with Long COVID.

Potential participants were recruited between August 2023 and November 2024 through ResearchMatch, a secure, publicly accessible online platform that connects volunteers with clinical studies at the NIH Clinical Center and academic medical centers across the United States [25]. Additional recruitment was conducted via in-person referrals from the Long COVID Clinic and affiliated peer support groups at Vanderbilt University Medical Center. Adults aged 18 years or older were eligible to participate if they met the following inclusion criteria: (1) experienced persistent symptoms with associated disabilities lasting longer than 3 months following an acute SARS-CoV-2 infection; (2) were employed prior to the infection; and (3) ever returned to work at any point after the infection. This study was approved by the Vanderbilt University Medical Center Institutional Review Board.

### Quantitative Phase

Individuals who expressed interest in the study completed a study inquiry form. A member of the research team subsequently contacted these individuals by telephone to conduct a prescreening assessment. Following confirmation of eligibility, the study procedures were explained in detail, and electronic informed consent was obtained. Eligible participants then received a secure REDCap link to complete a set of validated questionnaires. These instruments were designed to assess individual, interpersonal, community, and societal factors associated with sustained employment among individuals with Long COVID. The specific questionnaires utilized are described below.

**Employment outcomes** were evaluated using a questionnaire designed to collect data on participants’ employment status, job title, and a detailed description of their main job responsibilities before and after illness. The job title and job responsibilities were subsequently used to classify occupations according to the 23 major occupational groups defined by the U.S. Department of Labor in 2023 [26]. Additionally, an open-ended question prompted participants to describe their employment trajectory since acute SARS-CoV-2 infection. Sustained employment, conceptualized in this study as workforce attachment, was defined among participants who had returned to work as being engaged in paid employment at the time of assessment, irrespective of prior intermittent episodes of work cessation or work modifications. Participants classified as not sustaining employment were not engaged in paid employment at follow-up and did not report active or imminent plans to return to work. This definition differs from conventional occupational medicine definitions of sustained employment that emphasize uninterrupted continuous work over a fixed duration (e.g., ≥ 3 months) and was selected to better capture long-term workforce attachment in the context of chronic, fluctuating conditions such as Long COVID. Additionally, the validated Work Ability Index (WAI) was administered to participants employed at the time of survey completion to assess self-reported, perceived work ability. The WAI is a subjective measure capturing individuals’ perceptions of the balance between job demands and individual capacity, rather than an objective fitness-for-work assessment. It includes seven items, with a total score ranging from 7 to 49, where higher scores indicate better work ability. Work ability was classified as poor (7–27), moderate (28–36), good (37–43), and excellent (44–49) [27].

**The financial outcome** was assessed using the 11-item FACIT Measure of Financial Toxicity (FACIT-COST), which evaluates financial distress. Each item was rated on a five-point Likert scale (0 = not at all, 4 = very much), with lower scores indicating higher financial toxicity [28]. Financial toxicity was defined as a COST-FACIT score < 26 [29].

**Individual-level exposure variables** included sociodemographic factors (age, gender, race/ethnicity, education, insurance status, and primary income source). Physical function and cognitive ability were assessed using the Patient-Reported Outcomes Measurement Information System (PROMIS) Physical Function 8 and Cognitive Function 8, both of which are validated, patient-centered measurement tools. A T-score of 50 represents the average for the US general population; higher scores indicate better physical and cognitive function [30, 31]. Screening for post-traumatic stress disorder (PTSD) symptoms was measured using the PTSD Checklist for DSM-5 (PCL-5), with a cutoff of ≥ 50 suggesting probable PTSD diagnosis [32]. Depression symptoms were screened using the Beck Depression Inventory-II (BDI-II), with a cutoff of ≥ 20 suggestive of moderate to severe depression [33]. Fatigue was assessed using the Multidimensional Fatigue Inventory (MFI-20) [34], which includes five subscales (general, physical, mental, reduced activity, and reduced motivation), each comprising four items on a five-point Likert scale (range: 4–20), with higher scores indicating greater fatigue. A cumulative score exceeding 60 indicates significant fatigue [35]. Pain was measured using the EQ-5D-5L pain/discomfort item, with responses of “Moderate” or higher indicating the presence of pain [36]. Additional data were collected on acute SARS-CoV-2 infection, including dates and frequency of infections, as well as treatments administered. Information characterizing the symptomatology of Long COVID was also obtained.

**Relationship, community, and societal level exposure variables** were measured using a validated 25-item Craig Hospital Inventory of Environmental Factors (CHIEF) [37, 38]. The CHIEF assesses perceived environmental and contextual barriers that limit participation in life activities, including work participation, across interpersonal, community, and societal levels over the past 12 months. Participants were explicitly instructed to respond to CHIEF items with reference to barriers encountered in work participation or work-related activities. Each item is scored based on the frequency (0–4) and magnitude (1–2) of the barrier, producing an impact score from 0 to 8. Higher scores indicate a greater impact of barriers [37]. There is no established cutoff score for this questionnaire. However, mean CHIEF total scores (standard deviations) were as follows: 0.69 (0.87) for participants with disabilities, 0.89 (1.19) for participants with traumatic brain injury, and 1.25 (1.08) for those with spinal cord injury [38].

### Qualitative Phase

All participants who completed the quantitative phase were screened, and a subset was selected for the qualitative phase using a stratified purposeful sampling approach. Participants were stratified based on employment status into two groups: (1) those currently employed and (2) those who had returned to work but were unable to sustain employment. Semi-structured interviews were conducted to explore multilevel factors influencing employment sustainability. The interview guide was informed by the four-level aocial–ecological model, targeting individual, relationship, community, and societal determinants [23]. Topics included work-related challenges, employer accommodations, financial strain, workplace discrimination, and policy-level supports. All interviews were conducted by a master-prepared researcher via telephone, audio-recorded, transcribed verbatim and de-identified to ensure confidentiality. Verbal consent was reconfirmed before initiating each recorded interview. Data collection within each employment stratum continued until thematic saturation was achieved, defined as the point at which no new themes emerged. Consolidated Criteria for Reporting Qualitative Research (COREQ) guidelines were used to report the findings [39].

### Quantitative Analysis

Continuous variables were summarized using means and standard deviations (SD), while categorical variables were presented as counts and percentages. We then compared data using Student t-tests for continuous variables, and χ^2^ or Fisher exact tests, as appropriate, for categorical variables. All statistical analyses were performed using Stata version 17 (StataCorp, College Station, TX, USA).

### Qualitative Analysis

Qualitative data were analyzed using content analysis following the approach outlined by Elo and Kyngäs (2008) [40]. Transcripts were reviewed iteratively to facilitate immersion, and an initial coding framework was developed inductively while being informed deductively by the CDC’s four-level social-ecological model [23]. Two researchers independently performed open coding, followed by axial coding to refine categories and identify thematic relationships. A conceptual model was subsequently developed to illustrate the connections among themes, subthemes, and outcomes. Discrepancies in coding were resolved through discussion.

Integration of the quantitative and qualitative components followed a sequential explanatory approach. The quantitative component was developed and analyzed first, guiding the subsequent qualitative inquiry. Findings from both strands were merged through side-by-side comparison to assess convergence, complementarity, or divergence, with final interpretation informed by the integrated results.

## Results

### Quantitative Results

#### Sample Characteristics

A total of 184 individuals expressed interest in participating in the study; of these, 79 met eligibility criteria and completed the survey. The final sample comprised 79 individuals with Long COVID who were employed prior to SARS-CoV-2 infection—63 in full-time and 16 in part-time positions—and who subsequently returned to work. The mean (SD) duration from infection to survey completion was 1,077 (352) days. Participants had a mean (SD) age of 46 (12) years; 87% identified as White and 75% as female. Only 3% reported hospitalization during the acute phase of illness (Table 1). Prior to infection, participants were primarily employed in business and financial operations (*n* = 16), healthcare and related professions (*n* = 11), management (*n* = 10), administrative support (*n* = 9), and education (*n* = 8).

### Employment Status and Work Ability

**Fig. 1:**
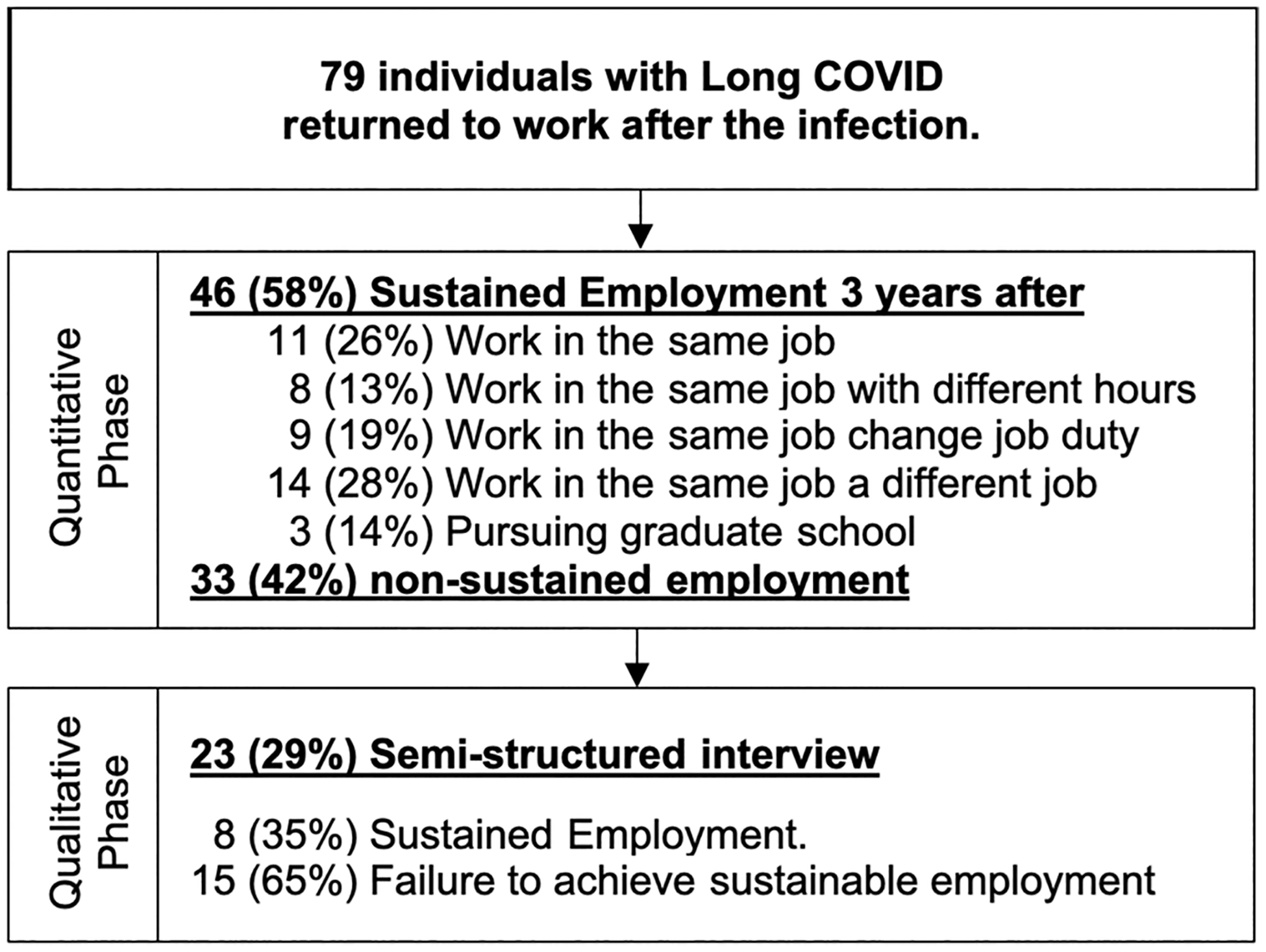
Flow diagram illustrating heterogeneity in sustained employment at follow-up, including continued workforce attachment with modified roles, hours, or job changes

Figure 1 displays employment outcomes among the 79 participants and illustrates heterogeneity in sustained employment, including continued workforce attachment with modified roles, hours, or job changes. At the time of the survey, 46 participants (58%), including three enrolled in graduate school, were employed. Among the 43 participants actively engaged in the workforce, the mean weekly working hours were 27.5 (SD = 3.5). Despite maintaining employment, 32 participants (75%) reported moderate to poor work ability, as assessed by the Work Ability Index (WAI). More than half expressed concerns about employment sustainability, with 23 (53%) fearing work cessation due to persistent Long COVID symptoms, 29 (67%) experiencing work-related challenges, and 19 (44%) needing additional support nearly 3 years post-infection.

Among the 33 participants who attempted to return to work but were unable to sustain employment, the vast majority (*n* = 32; 97%) expressed a desire to reenter the workforce, while acknowledging that return to work was not feasible due to health-related limitations. Key barriers to continued employment included physical limitations (*n* = 26; 72%), cognitive impairment (*n* = 20; 56%), and mental health challenges (*n* = 9; 25%).

### Factors Associated with Sustained Employment

Compared to participants with sustained employment, those unable to maintain employment demonstrated significantly lower physical function scores (33.9 vs. 38.8, *p* = 0.002), higher Charlson Comorbidity Index scores (2.3 vs. 1.0, *p* = 0.01), and greater utilization of physical therapy (76% vs. 48%, *p* = 0.02). They were also more likely to have consulted a social worker (36% vs. 13%, *p* = 0.03) and to have required home oxygen therapy (64% vs. 30%, *p* = 0.006) (Table 1).

Table 2 presents differences in environmental and attitudinal barriers between individuals with sustained versus non-sustained employment following illness. Participants with non-sustained employment reported significantly higher overall CHIEF scores compared to those with sustained employment (mean 2.3 vs. 1.6, *p* = 0.02), indicating greater cumulative exposure to environmental barriers. Non-sustained employment was associated with higher scores in the services and assistance (*p* = 0.01) and work/school barriers (*p* = 0.01) subscales. As described in the qualitative results (Themes 3–5, Table 3), workplace-related barriers reflected in the work/school subscale included constellations of workplace accommodations, organizational constraints, and workplace attitudes associated with recurrent work disruption and difficulty sustaining employment over time.

### Financial Outcome

Prior to infection, 91% of participants identified wages as their primary source of income, which declined to 46% at the time of survey completion. Among those without sustained employment, income was primarily derived from support from others (30%), disability benefits (21%), and savings or investments (18%). Financial toxicity was common, with 87% of participants reporting burden, with a mean FACITCOST score of 13.1 (SD = 9.4). Participants without sustained employment had significantly lower scores compared to those with sustained employment (10.4 vs. 15.0, *p* = 0.03), reflecting greater financial burden.

### Qualitative Results

**Fig. 2:**
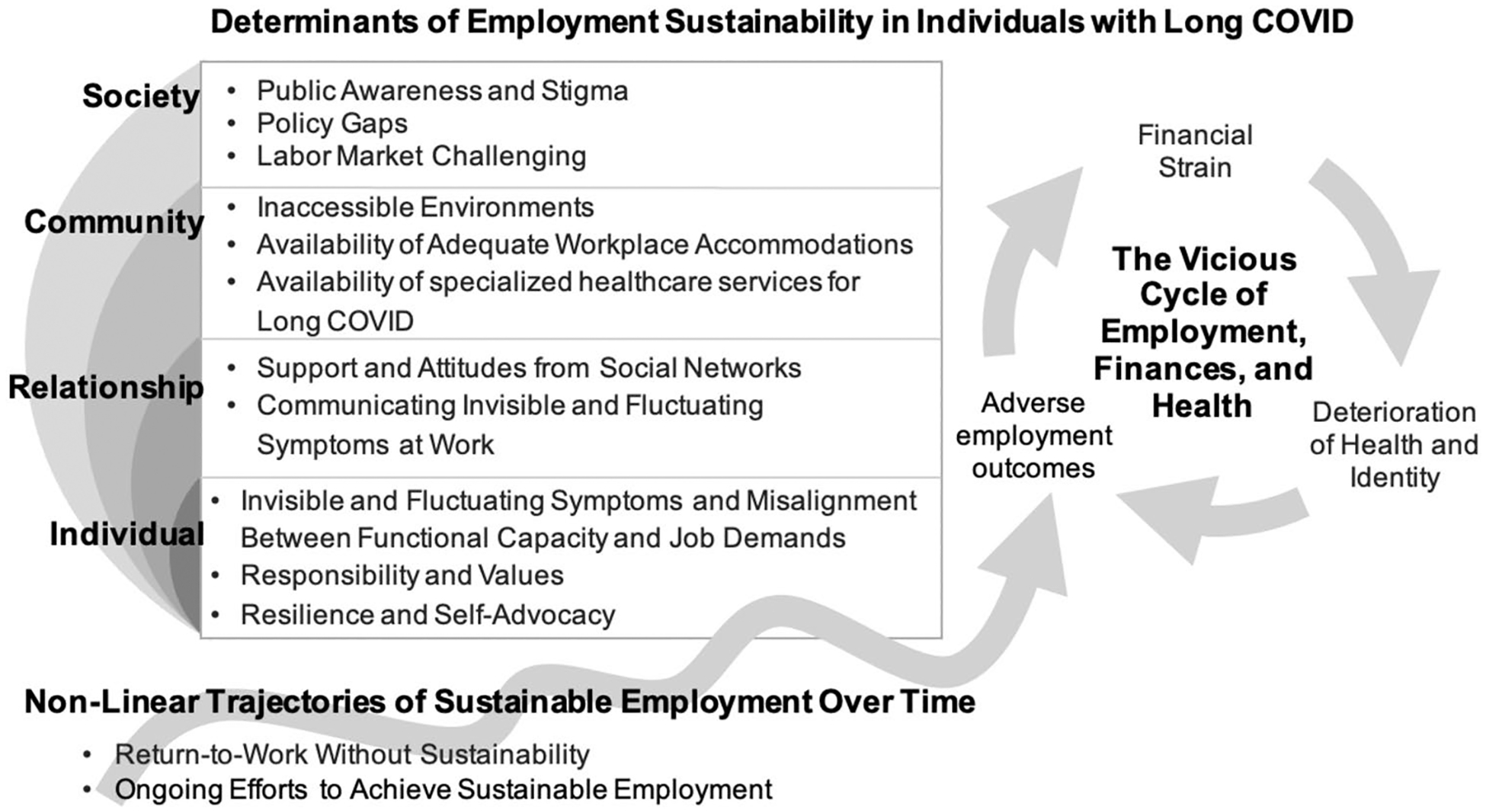
Conceptual model of 6 key themes identified in the study and their relationship Employment sustainability following initial return to work among individuals with Long COVID is nonlinear, encompassing (1) return without sustained employment and (2) continued efforts to achieve stable employment. These patterns are influenced by multilevel determinants at the individual, interpersonal, community, and societal levels. Adverse employment outcomes may exacerbate financial strain, undermine health and identity, and further hinder employment sustainability. Bold text = themes; plain text = subthemes; the fluctuating arrow represents the nonlinear trajectory of employment sustainability after initial return to work

We conducted a qualitative analysis of 79 open-ended survey responses detailing participants’ employment trajectories following acute SARS-CoV-2 infection, supplemented by semi-structured interviews with 23 participants. Of those interviewed, 8 had sustained employment (including one enrolled in graduate school), while 15 had returned to work but were unable to maintain employment. Interviews lasted 45–60 min. Six overarching themes emerged from the analysis, organized into three domains: (1) Return-to-Work Trajectories, (2) Multilevel Determinants of Long-Term Employment Sustainability, and (3) Consequences of Adverse Employment Outcomes. These findings provide contextual depth and explanatory insight to complement the quantitative results. A conceptual model illustrating the relationships among themes, subthemes, and employment outcomes is shown in Fig. 2. A summary of themes, subthemes, codes, and illustrative quotations is presented in Table 3.

### Domain 1: Return-to-Work Trajectories Among Participants with Long COVID

#### Theme 1: Nonlinear Trajectories of Sustainable Employment Over Time

The employment experiences of participants with Long COVID in this analysis were characterized by continuous adaptations and challenges. Two subthemes emerged, reflecting distinct employment patterns. *Return-to-Work Without Sustainability* describes participants who resumed employment but were unable to maintain long-term job stability despite their efforts. These participants frequently cycled between workforce reentry and medical leave, ultimately resulting in workforce disengagement. In contrast, *Ongoing Efforts to Achieve Sustainable Employment* describes participants who made continuous attempts to remain employed by modifying their work hours, duties, or career paths (including changing jobs within or outside their original field). Despite these efforts, some participants continued to encounter persistent challenges, leaving them vulnerable to future job instability or disengagement (Table 3).

### Domain 2: Multilevel Determinants of Long-Term Employment Sustainability in Participants with Long COVID

Sustained employment following return to work serves as a critical indicator of successful work reintegration, shaped by a complex interplay of factors at the individual, relationship, community, and societal levels. Four key themes emerged, illustrating these multilevel influences (Table 3).

#### Theme 2: Individual Determinants of Employment Sustainability

Qualitative findings revealed that individual-level determinant encompasses biological and individual-level factors that contribute to the likelihood of achieving sustainable employment [23]. Three subthemes emerged. First, *Invisible and Fluctuating Symptoms and Misalignment Between Functional Capacity and Job Demands* highlights how persistent fatigue, cognitive deficits, and physical limitations—coupled with the unpredictable nature of symptoms—undermined participants’ ability to fulfill essential job duties and compromised employment sustainability. Second, *Responsibility and Values* captures how a sense of duty to family, coworkers, or clients served as a motivator for sustained employment. However, this same sense of responsibility may serve as both a facilitator and a barrier, as it can contribute to anxiety about underperformance or causing harm to others. Finally, the subtheme of *Resilience and Self-Advocacy* reflects participants’ adaptability in navigating career setbacks related to Long COVID, including acceptance of lower-paying positions, pursuing self-employment for greater flexibility, or stepping down from leadership positions. Concurrently, these participants engaged in proactive efforts to obtain workplace accommodations to support the management of persistent health challenges (Table 3).

#### Theme 3: Relationship Determinants of Employment Sustainability

Relationship-level determinants explore the influence of close interpersonal relationships on the risk of impaired employment sustainability [23]. Two subthemes emerged. *Support and Attitudes from Social Networks* emphasize the critical role of support from family, friends, healthcare providers, coworkers, and supervisors. Family members provided emotional support and practical advice, while friends in the same field offered guidance on workplace issues, and healthcare providers recommended flexible work options. Although coworkers and supervisors were generally supportive, misunderstandings often occurred due to fluctuating and diverse symptoms, as well as conflicting performance expectations related to health needs. The second subtheme, *Communicating Invisible and Fluctuating Symptoms at work*, captures how participants with invisible, fluctuating symptoms struggle to communicate their limitations, especially when temporary improvements are mistaken for full recovery. This can lead to repeated justifications of their condition to supervisors and colleagues (Table 3).

#### Theme 4: Community Determinants of Employment Sustainability

Community-level determinants examined workplace and neighborhood environments that influence the sustainability of employment [23]. Three subthemes emerged: *Inaccessible Environments* highlights how the workplace surroundings and natural environment, as described by participants, such as long hallways and stairs, extreme temperatures, and loud noise, increased physical and cognitive exertion and exacerbated symptoms, thereby hindering job performance and leading to fatigue, stress, and reduced productivity, which increased the risk of work disengagement. *Availability of Adequate Workplace Accommodations* emphasizes the importance of accommodations, such as flexible schedules or remote work, in supporting participants with Long COVID. While these accommodations are generally implemented with minimal resistance, requests for changes to the physical workspace layout or broader organizational adjustments were more difficult to obtain. Negative employer attitudes, limited awareness of Americans with Disabilities Act (ADA) provisions, and bureaucratic obstacles within organizational accommodation request processes significantly impede timely access to appropriate workplace supports. Third, *Availability of specialized healthcare services for Long COVID*: limited availability of appropriate medical care presents an additional barrier to sustained employment, as it affects participants’ ability to manage ongoing symptoms and maintain functional capacity at work (Table 3).

#### Theme 5: Societal Determinants of Employment Sustainability

Societal-level determinants encompass broad factors affecting employment sustainability, including social and cultural norms, economic and labor market conditions, and policy frameworks [23]. Three subthemes emerged. First, *Public Awareness and Stigma* reflects how limited societal understanding of Long COVID fosters stigma, diminishes empathy, and reduces access to workplace accommodations and healthcare, ultimately undermining employment sustainability. Second, *Policy Gaps*, including restrictive disability benefit eligibility criteria, prolonged waiting periods, Family and Medical Leave Act (FMLA) exemptions for small businesses, and inconsistent state-level protections, create substantial barriers for participants with Long COVID, exacerbating financial and employment instability. Third, *Labor Market Challenges* captures the broader economic context, including pandemic-related workforce reductions, constrained rehiring opportunities, and the shift toward lower-paid or less specialized roles. These conditions disproportionately affected participants with ongoing health impairments – particularly those requiring remote work or other accommodations (Table 3).

### Domain 3: Consequences of Adverse Employment Outcomes in Participants with Long COVID

#### Theme 6: The Vicious Cycle of Employment, Finances, and Health

Failure to sustain employment had significant consequences for both financial stability and health outcomes. Two subthemes emerged. *Financial strain*, resulting from job loss, reduced income, and increased healthcare costs, forced participants to deplete savings and accrue debt. While temporary support from family or friends provided short-term relief, it was often unsustainable. Efforts to access public assistance were frequently obstructed by bureaucratic barriers, such as income thresholds for SSDI (Social Security Disability Insurance) and the absence of a standardized diagnostic label for Long COVID-related disability. Even when benefits like SSDI were secured, they often fell short of covering basic living expenses and came with trade-offs that further diminish overall income. Second, the *Deterioration of Health and Identity* highlighted how financial strain worsened existing health conditions and limited access to necessary care due to cost and insurance limitations. These challenges, combined with the inability to contribute financially, led to emotional distress and identity loss (Table 3).

### Integration of Quantitative and Qualitative Components

Integration of the quantitative and qualitative components demonstrated convergence across methods and across multiple levels of influence. Quantitative findings identified significant differences between participants with sustained and non-sustained employment in individual health status, environmental and workplace barriers, and financial burden, while qualitative data elucidated how these factors interacted across individual health limitations and financial strain, interpersonal support and communication, workplace accommodations and organizational constraints, and broader societal and policy contexts. Together, these findings support interpretation of employment sustainability following Long COVID as shaped by cumulative, multilevel influences rather than isolated individual or workplace factors.

## Discussion

In this mixed-methods study of 79 individuals with Long COVID who initially returned to work following acute infection, slightly more than half remained employed at up to 3 years post-infection. Among those still employed, the majority reported reduced work capacity and uncertainty regarding the sustainability of their employment. Participants no longer employed at follow-up demonstrated significantly greater physical, cognitive, and psychological impairment, higher comorbidity and healthcare utilization, and more severe financial hardship. Together, these findings confirm that returning to work after Long COVID does not equate to employment recovery. Instead, employment sustainability emerges as a distinct outcome shaped by cumulative, multilevel barriers. Qualitative findings revealed that return-to-work trajectories were often nonlinear and influenced by a complex interplay of barriers operating at the individual, interpersonal, community, and societal levels. These barriers included fluctuating and invisible symptoms (which contribute to frequent reports of being dismissed by employers and health care professionals), occupational mismatches, inadequate social and environmental supports, limited workplace accommodations, restricted access to specialized care, pervasive stigma, policy gaps, and labor market constraints. Failure to sustain employment was closely associated with worsening health and financial hardship, with loss of employment commonly linked to increased financial strain, declining health, and a diminished sense of identity and purpose. These findings highlight the complex challenges faced by Long COVID patients that impair quality of life and independence.

Similar to the current era impact of COVID, most participants in our study were not hospitalized during acute infection; this cohort reflects substantial illness burden. Nearly half screened positive for probable post-traumatic stress disorder. Rather than indicating isolated psychiatric vulnerability, the high prevalence of PTSD symptoms likely reflects prolonged symptom burden, uncertainty during recovery, fragmented care, and repeated dismissal by employers or clinicians—experiences known to contribute to traumatic stress. Future longitudinal studies integrating detailed measures of symptom trajectories, healthcare experiences, and workplace exposures are needed to elucidate the pathways linking employment sustainability and mental health outcomes, including PTSD, among individuals with Long COVID. Similarly, the need for home oxygen in a largely non-hospitalized sample underscores that clinically significant physiological impairment can occur outside inpatient settings. Together, these findings reinforce that poor work sustainability following Long COVID is not confined to severe acute hospitalization but importantly affects individuals with persistent, functionally limiting illness managed in outpatient contexts.

Notably, participants who were unable to sustain employment were, on average, in their mid-40 s, reflecting prime working age rather than proximity to retirement. This demographic profile is comparable to findings from international Long COVID employment studies, underscoring that workforce disengagement is occurring among core members of the labor force rather than marginal or late-career workers. In the United States, where nearly one in seven working-age adults is estimated to be affected by Long COVID, the loss of employment among individuals in their prime working years represents a substantial public health and economic concern.

Our data extend previous research by underscoring the ongoing vulnerability of sustained employment among individuals with Long COVID [11, 12, 41–44]. Notably, 42% of participants who initially returned to work following SARS-CoV-2 infection were no longer employed nearly three years later. Even among those who remained in the workforce, more than half expressed concerns about their capacity to maintain their occupational roles. These findings highlight the persistent impact of Long COVID on occupational functioning and reinforce that return-to-work status, while a critical milestone is an insufficient proxy for full recovery or durable functional reintegration. Longitudinal monitoring of employment trajectories is essential to identify individuals at heightened risk of job loss or reduced work capacity.

Evidence from occupational rehabilitation and vocational health literature indicates that the probability of sustained return to work declines as work absence duration increases, with critical inflection points often observed beyond approximately six weeks and again at six months [45]. Prolonged absence is associated with elevated risk of long-term work disability, particularly in the absence of coordinated vocational intervention. In this context, the observed employment loss nearly three years following acute SARS-CoV-2 infection may reflect the combined effects of persistent symptom burden, fluctuating functional capacity, and variable access to timely, coordinated clinical and occupational support. Although SARS-CoV-2 transmission rates have varied over time, reinfection remains possible, and epidemiologic evidence suggests that repeated infections may be associated with cumulative health risk [46], which could plausibly influence long-term employment sustainability. Together, these findings underscore the importance of early, work-focused clinical management and integrated vocational support to mitigate prolonged work disruption and reduce the risk of long-term workforce disengagement, alongside ongoing efforts to prevent infection and support overall health stability.

Given the marked clinical heterogeneity of Long COVID, treatment approaches must be individualized and guided by clinical phenotype rather than standardized rehabilitation protocols. This study found that sustained employment among individuals with Long COVID is shaped by a complex interplay of individual, interpersonal, community, and societal factors. Persistent symptoms—such as fatigue, cognitive dysfunction, and mental health conditions—commonly impair occupational functioning, mirroring patterns observed in other chronic conditions like cancer and traumatic injury [47, 48]. However, Long COVID poses unique challenges due to its relapsing–remitting course [4, 5], the invisibility and heterogeneity of symptoms [3–5], and the absence of a definitive diagnostic standard [49]. A growing body of evidence indicates that many individuals with Long COVID experience post-exertional malaise or symptom exacerbation—characterized by fluctuating physical, cognitive, or systemic symptoms following exertion [11]—which may contribute to fluctuating work capacity and difficulty sustaining employment. Although our measures cannot diagnose post-exertional malaise as a discrete clinical construct, our study documented substantial and fluctuating fatigue as well as physical and cognitive impairment—symptom domains commonly implicated in post-exertional malaise. Accordingly, we cannot determine the specific contribution of post-exertional malaise in this cohort. Instead, exertion-related symptom exacerbation is discussed as one potential mechanism that may contribute to the symptom burden observed and to the nonlinear employment trajectories identified. In this context, interventions requiring structured or graded exercise may exacerbate symptoms and be harmful for individuals with ME/CFS-like features.

Interprofessional models integrating longitudinal symptom monitoring, pacing, and energy-management strategies, and individualized workplace accommodations may therefore be more appropriate than standardized rehabilitation approaches. Behavioral interventions, including cognitive behavioral therapy (CBT), have primarily been examined as supportive approaches to facilitate coping, adjustment, and self-management of fluctuating and persistent symptoms—such as fatigue and mood-related distress—in individuals with Long COVID, rather than as disease-modifying treatments [50, 51]. While some structured rehabilitation programs, including online CBT and integrative physical–mental health approaches, have been associated with improvements in fatigue, concentration, and quality of life [52], these findings should be interpreted cautiously. At present, pharmacologic, dietary, and device-based interventions lack robust evidence of efficacy in this population [52], underscoring the need for phenotype-informed, mechanism-aware approaches to support sustainable participation in work and daily life.

Compared to individuals with Long COVID who sustained employment, those unable to maintain employment reported significantly greater cumulative exposure to environmental barriers, as reflected by higher overall CHIEF scores. While item-level differences were examined and are presented in Table 2 for descriptive transparency, they were not interpreted independently, as statistically robust inference is strongest at the total and subscale levels per the CHIEF’s design and validation [37]. Differences were most pronounced—and statistically significant—in the services and assistance and work/school subscales, indicating challenges related to accessing healthcare, support services, and workplace accommodations. Mean subscale scores across CHIEF domains were consistently higher among participants with non-sustained employment, even though not all subscales reached statistical significance, suggesting a pattern of cumulative environmental disadvantage rather than isolated workplace obstacles. The level of environmental barriers observed in this cohort, as measured by the CHIEF, was comparable to or higher than levels reported in prior studies of individuals with spinal cord injury, traumatic brain injury, and other disability populations [38]. Together, these findings highlight the presence of substantial structural barriers and underscore the need for targeted interventions and policy reforms to support vocational stability among individuals with Long COVID.

From a public health policy perspective, the long-term occupational consequences of Long COVID necessitate its formal integration into chronic disease management frameworks and a reexamination of US federal disability determination processes [53]. Existing Social Security Disability Insurance (SSDI) eligibility criteria, which rely on objective diagnostic evidence, are often misaligned with the clinical presentation of Long COVID [54]. Drawing from lessons in conditions such as Myalgic Encephalomyelitis/Chronic Fatigue Syndrome (ME/CFS) and Fibromyalgia, where symptom invisibility and diagnostic ambiguity similarly pose barriers, policymakers can develop more equitable assessment approaches [54]. Access to care is further hindered by the 24-month Medicare eligibility waiting period for SSDI recipients, delaying treatment for individuals under age 65 with Long COVID-related disability. Legislative reform in this area could significantly improve timely healthcare and reduce downstream health and economic burdens for participants under 65 with Long COVID-related disability [55]. In addition, updates to the implementations of the Americans with Disabilities Act (ADA) are warranted to reflect the fluctuating and episodic nature of Long COVID-related impairments. Current ADA guidance provides insufficient guidance on workplace accommodations, resulting in inconsistent application and increased risk of workplace discrimination. Policy revisions should emphasize functional impairments, rather than diagnostic certainty, as the basis for accommodation eligibility, along with expanding employer education on supporting workers with intermittent or nonvisible disabilities [56]. Finally, sustained public investment in comprehensive Long COVID care—including multidisciplinary clinical services, vocational rehabilitation, mental health support, clinician education, public awareness campaigns, and employer training—is essential to support long-term recovery and employment sustainability among those with Long COVID [57].

This study has several notable strengths. By employing a mixed-methods design and extending the follow-up period beyond that of prior research [11, 12, 41–44], it captures the dynamic and evolving nature of post-COVID employment trajectories. The use of a social-ecological framework further enables a nuanced analysis of the multilevel factors—individual, interpersonal, community, and societal domains—that influence sustained employment among individuals recovering from Long COVID. However, several limitations should be considered when interpreting our findings. First, the study sample was drawn from participants who had access to an in-person post-COVID clinic or were recruited online through ResearchMatch, which may introduce selection bias, as these participants may be more motivated to seek care or participate in research to better understand their condition. Second, many participants reported experiencing multiple and severe symptoms, which may limit the generalizability of our findings to those with milder or transient forms of Long COVID. Third, Long COVID status was based on self-report without confirmation of a prior COVID-19 diagnosis, which may affect diagnostic accuracy. Fourth, individuals with severe acute COVID-19 were underrepresented in the sample, with only 3% having been hospitalized. Fifth, neither the CHIEF nor the Work Ability Index (WAI) is designed to function as a workplace or occupational assessment tool or to provide objective evaluations of specific job tasks, workplace conditions, or individualized fitness-for-work. Instead, both measures capture participants’ perceived environmental barriers and work capacity, and findings should be interpreted within this scope, acknowledging that the applicability of these self-reported measures may vary across occupational contexts, healthcare systems, and countries. Sixth, although workplace-related barriers and supports emerged in both quantitative and qualitative findings, the study was not designed to conduct a comprehensive or employer-level workplace analysis. Finally, symptom experiences were assessed via self-report, which may be subject to recall bias or subjective interpretation.

Future research should prioritize identifying early predictors of non-sustained employment and determining the optimal timing and sequencing of clinical and vocational interventions for individuals with Long COVID. Prospective studies are needed to evaluate when work-focused rehabilitation, occupational health involvement, and coordinated multidisciplinary care are most effective in preventing prolonged work disruption. In addition, future investigations should examine how specific job demands, task characteristics, and occupational contexts interact with fluctuating symptom patterns to influence long-term employment sustainability. Greater integration of primary care, rehabilitation services, and workplace-based support may help mitigate extended absence and reduce the risk of long-term workforce disengagement, thereby limiting downstream financial and health consequences.

## Conclusion

This study highlights the substantial and enduring long-term employment challenges faced by individuals with Long COVID, with more than two in five of those who initially returned to work experiencing job loss nearly three years post-infection. Even among those who remained employed, many expressed concerns regarding their work capacity. Long COVID’s impact on employment sustainability is shaped by a complex interplay of fluctuating symptoms, misalignment between functional capacity and job demands, inadequate workplace accommodations, stigma, policy gaps, and broader labor market challenges. Interprofessional care and behavioral interventions are crucial for supporting recovery, while policy changes—including revisions to disability determination processes and the Americans with Disabilities Act—are necessary to improve access to care and workplace protections. Sustained public investment in research, effective clinical treatments, comprehensive care infrastructure, and policy reform is critical to promoting sustainable employment.

## Figures and Tables

**Table 1:** Individual-level exposure stratified by employment outcome

	All (*n* = 79)	Non-sustained employment (*n* = 33)	Sustained employment (*n* = 46)	*p* ^€^
Individual-level factors level exposure				
Age, Mean (SD), y	46.1 (12.3)	48.6 (12.2)	44.0 (12.1)	0.1
Female, *n* (%)	59 (75)	22 (68)	37 (80)	0.29
White race, *n* (%)	69 (87)	28 (85)	41 (89)	0.73
Hispanic ethnicity, *n* (%)	5 (6)	2 (6)	3 (7)	0.99
Married and/or Living with Significant Other/Partner, *n* (%)	47 (60)	20 (61)	27 (59)	0.99
Education (≥ bachelor’s degree), *n* (%)	61 (77)	22 (67)	39 (85)	0.10
Primary source of income after infection, *n* (%)				
Work	36 (46)	0(0)	36(68)	< 0.001
Pension or retirement benefits	1 (1)	1(3)	0(0)	
Disability benefits from work	7 (9)	7(21)	0(0)	
SSDI	5 (6)	3(9)	2(4)	
Social Security Supplement Security Income	2 (3)	2(6)	0(0)	
Personal savings or investments	8 (10)	6(18)	2(4)	
Support by other(s)	12 (15)	10(30)	2(4)	
Other	8 (10)	4(12)	4(9)	
Uninsurance, *n* (%)	2 (3)	1 (3)	1(2.2)	0.99
Charlson Comorbidity Index, Mean (SD)	1.5 (2.3)	2.3 (3.1)	1.0 (1.3)	0.01
Admitted to hospital due to COVID-19, *n* (%)	5 (7)	3 (9)	2 (5)	0.5
Physical function, Mean (SD)	36.8 (7.3)	33.9 (7.0)	38.8 (6.8)	0.002
Cognitive function, Mean (SD)	32.4 (8.3)	31.2 (7.6)	33.3 (8.8)	0.26
PTSD, *n* (%)	34 (43)	17 (52)	17 (37)	0.25
Moderate to severe Depression, *n* (%)	49 (67)	23 (79)	26 (59)	0.08
Significant fatigue, *n* (%)	74 (94)	31 (94)	43 (94)	0.99
Pain, *n* (%)	49 (63)	24 (73)	25(56)	0.16
Duration from COVID-19 infection to survey completion, Mean (SD) (day)	1044 (381)	1055 (388)	1036 (380)	0.83
Hospital admission for COVID, *n* (%)	5 (7)	3 (9)	2 (4)	0.65
Healthcare utilization post-infection, *n* (%)				
Emergency department	57 (72)	23 (70)	34 (74)	0.80
Hospitalization	16 (20)	9 (27)	7 (15)	0.26
Specialist or primary care	76 (96)	32 (97)	44 (96)	0.99
Rehabilitation facility	3 (4)	0 (0)	3 (7)	0.26
Physical therapy	47 (60)	25 (76)	22 (48)	0.02
Speech therapy	21 (27)	11(33)	10 (22)	0.31
Occupational therapy	26 (33)	14 (42)	12 (26)	0.15
Mental health	53 (67)	21 (64)	32 (70)	0.63
Social worker	18 (23)	12 (36)	6 (13)	0.03
Vocational rehabilitation counselor	8 (10)	5 (15)	3 (7)	0.27
Home oxygen	35 (44)	21 (64)	14 (30)	0.006

* Data presented as *n* (%) unless otherwise noted and may not add to 100% due to rounding variables

€ Calculated by student T test for continuous variables, Kruskal–Wallis for count data, and χ2 or Fisher’s exact tests, as appropriate, for categorical variables.

*SSDI* = social security disability insurance

**Table 2:** Relationship, community, and societal level exposure stratified by employment outcome

	All (*n* = 79)	Non-sustained employment (*n* = 33)	Sustained employment (*n* = 46)	*P* ^€^
**CHIEF total score**	**1.9 (1.4)**	**2.3 (1.6)**	**1.6 (1.3)**	**0.02**
**Physical/structural barriers subscale**	**2.1 (1.6)**	**2.4 (1.7)**	**1.8 (1.9)**	**0.07**
Design and layout of your home	1.5 (2.3)	1.7 (2.1)	1.3 (2.4)	0.49
Design and layout of your workplace or school	2.0 (2.6)	2.3 (2.3)	1.9 (2.7)	0.65
Design and layout of your community	1.4 (2.2)	1.7 (2.1)	1.2 (2.2)	0.35
Natural environment—temperature, climate, terrain	3.4 (2.9)	3.6 (3.1)	3.2 (.6)	0.51
Other aspects of your surroundings—lighting, noise, crowds	3.6 (3.2)	4.1 (3.1)	3.3 (3.2)	0.25
Lack of computer technology	0.5 (1.6)	1.0 (2.4)	0.9 (0.4)	0.03
**Attitudes and support barriers subscale**	**1.5 (1.7)**	**1.8 (1.9)**	**1.2 (1.5)**	**0.06**
Other people’s attitudes at home	1.6 (2.4)	2.0 (2.8)	1.2 (2.0)	0.10
Other people’s attitudes in the community	1.2 (1.9)	2.0 (2.2)	0.6 (1.4)	0.002
Lack of support and encouragement from others in home	1.6 (2.5)	1.8 (2.8)	1.5 (2.3)	0.30
Lack of support and encouragement from others in community	1.4 (2.2)	1.9 (2.3)	1.0 (2.3)	0.03
Prejudice or discrimination	1.7 (2.4)	1.6 (2.4)	1.8 (2.4)	0.33
**Services and assistance subscale**	**1.6 (1.8)**	**2.1 (1.8)**	**1.2 (1.5)**	**0.01**
Availability of transportation	1.5 (2.7)	2.3 (3.3)	1.0 (2.2)	0.06
Access to information in a usable or understandable format	1.7 (2.7)	2.0 (2.6)	1.6 (2.7)	0.48
Availability of education and training	1.1 (2.1)	1.2 (2.1)	1.0 (2.1)	0.82
Availability of healthcare services/medical care	2.8 (2.7)	3.5 (2.9)	2.2 (2.5)	0.02
Lack of personal equipment or special devices	1.4 (2.5)	2.1 (2.9)	0.9 (2.1)	0.03
Lack of someone else’s help in the home	1.5 (2.3)	1.9 (2.6)	1.2 (2.0)	0.08
Lack of someone else’s help in the community	1.0 (1.9)	1.6 (2.1)	0.6 (1.7)	0.02
**Policies subscale**	**2.4 (2.3)**	**2.8 (2.6)**	**2.1 (2.2)**	**0.11**
Lack of programs or services in the community	2.2 (2.8)	2.6 (2.7)	1.9 (2.9)	0.17
Policies and rules of businesses/organizations	2.2 (2.9)	2.3 (3.1)	2,1 (2.8)	0.39
Education/employment programs and policies	2.6 (3.1)	3.8 (3.5)	2.3 (3.0)	0.09
Government programs and policies	2.6(3.0)	3.3 (3.1)	2.1 (2.9)	0.05
**Work/school barriers subscale**	**2.1 (2.4)**	**3.6 (2.9)**	**1.7 (2.2)**	**0.01**
Lack of someone else’s help at work or school	1.6 (2.6)	3.1 (3.3)	1.3 (2.3)	0.03
Other people’s attitudes at school or work	2.3 (2.6)	3.9 (3.1)	2.1 (2.5)	0.05
Lack of support and encouragement from others at school or work	2.0 (2.6)	4.0 (3.2)	1.6 (2.4)	0.01

* Data presented as mean (standard deviation)

€ Calculated by student T test for continuous variables, Kruskal–Wallis for count data, and χ2 or Fisher’s exact tests, as appropriate, for categorical variables

‡ There is no established cutoff score for Craig Hospital Inventory of Environmental Factors. However, mean CHIEF total scores (standard deviations) were as follows: 0.69 (0.87) for individuals with disabilities, 0.89 (1.19) for individuals with traumatic brain injury, and 1.25 (1.08) for those with spinal cord injury.^53^

**Table 3:** Themes, subthemes, and illustrative quotes

Domain 1: return-to-work trajectories among participants with long COVID	
Theme 1: Non-linear trajectories of sustainable employment over time	
Subtheme	Illustrative quotes
Return-to-work without sustainability	I tried [home improvement store] part-time, then [retail store] full-time, but neither worked—now on medical leave for COVID-19 (P 10)
	I took an insurance job but struggled with physical issues. Despite using intermittent FMLA [unpaid medical leave] and reducing hours, I lost my job (P 73)
	I returned to work in August 2021, but worsening symptoms led to a panic attack and flare-up—I resigned after 2.5 years (P 76)
	After the first infection in 2020, I worked through significant health struggles. Once on the right medications, exercise intolerance and asthma worsened. Two subsequent infections left me missing enough work that I was encouraged to apply for disability (P 6)
Ongoing efforts to achieve sustainable employment	I returned to work a year post-infection, same employer, slightly different role (P 43)
	Still in the same job, but 4 years later, I can’t function like I used to (P 39)
	As my symptoms worsened, I worried about my ability to perform at work and whether my boss would be flexible or let me go (P 45)
	It’s not linear. There have been times when I could work a bit more and months when I could not work at all. There have been periods of recovery and periods of sudden and lengthy worsening. It has now been over 4.5 years, and I am still substantially limited (P 34)
Domain 2: Multilevel determinants of long-term employment sustainability in participants with long COVID	
Theme 2: Individual determinants of employment sustainability in long COVID
Subtheme	Illustrative quotes
Invisible and fluctuating symptoms and misalignment between functional capacity and job demands	*Code: The discrepancy between an individual’s capabilities and the demands of their job*
	I couldn’t lift anymore and struggled with basic tasks, like walking to assist patrons. I’d often mix up lists, get event dates wrong, and make mistakes entering information (P 58)
	It’s difficult to multitask, which is required of the job or any job (P 16)
	I tried going back to work, but during class, I’d end up struggling to breathe (P 21)
	*Code: Invisible and fluctuating symptoms*
	I’ll sign up for assignments thinking I can handle it, but with long COVID, you never know how you’ll feel day to (P 53)
	Today’s a good day, no problems. But tomorrow? Who knows. Every day is different, and no day feels the same. I never know how I’ll wake up in the morning (P 21)
	It’s invisible, no one can see it unless I tell them (P 22)
Responsibility and values	*Code: Responsibility to others*
	I want to work full-time as long as I can, especially financially to help out my household (P 45)
	I was trying to get back to work to relieve my substitute [teacher] and have normalcy for my preschooler [students] (P 64)
	Knowing I have a lot of people that depend on me (P 71)
	*Code: Work ethic*
	I knew I shouldn’t have gone, because doing it wrong could put people’s lives at risk (P 2)
Resilience and self-advocacy	*Code: Resilience*
	I took a step back and started working at my chiropractor’s front desk for $15 an hour. I went from making $150 K a year to that (P 14)
	I started my own business, hoping it would give me the flexibility to attend doctor appointments and manage other things (P 75)
	I returned to work without any assistance and tried to do as much as I could. As my health and performance declined, I was removed from my leadership position (P 66)
	*Code: Self-advocacy*
	And really had to adjust with work and working with my employer and my boss about what I can and can’t do (P 45)
Theme 3: Relationship determinants of employment sustainability in long COVID
Subthemes	Illustrative quotes
Support and attitudes from social networks	*Code: Family and friend*
	My family is very supportive—my parents, in-laws, and siblings suggest ideas like asking for more breaks, using FMLA [unpaid medical leave], or finding ways to get extra paid time off, helping me figure out what to ask my boss and HR (P 45)
	I have a close group of college friends who work in the same field and know my coworkers. They check in regularly, ask about work, and even offer to talk to my boss if needed (P 45)
	*Code: Healthcare provider*
	After discussing work with my long COVID doctor, who suggested using intermittent FMLA [unpaid medical leave] if I’m not reducing hours, I’ve been trying to pace myself while working full-time with more flexibility (P 45)
	*Code: Co-worker and supervisor*
	If I mention having a bad day, they might handle an email I’d normally take or delay assigning a project until I can discuss it (P 4)
	My supervisor initially reduced my workload but slowly piled it back on. The stress caused memory issues and anxiety, which frustrated both of us. After a harsh conversation and a panic attack, I resigned (P 76)
Communicating invisible and fluctuating symptoms at work	*Code: Communicating invisible symptom*
	Only others with chronic invisible diseases truly understand what it’s like (P 23)
	*Code: Communicating fluctuating symptom*
	The hardest part is repeatedly saying, “I’m not doing well—I can’t do this.” Some co-workers think if I’m having a better day, things are improving, but then when I have a bad day and struggle with brain fog, they’re surprised and ask, “What happened?” (P 45)
Theme 4: Community determinants of employment sustainability in long COVID
Subthemes	Illustrative Quotes
Inaccessible environments	The elevator was broken, and I can’t do stairs anymore—not even halfway (P 58)
	We have four long hallways, and it’s really taxing—I get out of breath (P 45)
Availability of adequate workplace accommodations	*Code: Types of accommodations as determinants of access to appropriate support*
	I’ve asked the person I sublease from if we can add a door or wall—something. But nothing’s changed. I hear hairdryers and music all day, and by 5:00, I’m angry (P 75)
	My accommodations include recording meetings, working from home 1–2 days a week, and flexible paid time off for doctor’s appointments (P 2)
	*Code: Employer attitudes, knowledge, and implementation process of ADA regulations*
	I requested an accommodation on July 1 to limit outdoor time due to heat sensitivity. The process felt invasive—more like an interrogation—they even contacted my doctor without consent. Still, I was assigned to an outdoor event, though an indoor option exists. I’ve followed up, but there’s been no response (P 2)
	I was on intermittent FMLA [unpaid medical leave] for about a year and a half toward the end of my time there. I was supposed to be working 28 h (per week), but my employers kept giving me 40–50 h’ worth of work. I tried to take some unpaid time off, but they wouldn’t allow it. (P 58)
	I asked about a temporary reassignment, and my boss said no unless I took a big pay cut. Later, I found out FMLA [unpaid medical leave] allows it without a cut—she steered me wrong (P 76)
Availability of specialized healthcare services for long COVID	[A] lot of these appointments I must make three, six months in advance to get in (P 45)
	It was hard to get help. In Florida, I couldn’t get any, even though my doctors tried to refer me to a clinic in Miami. Then I moved to Birmingham (Alabama) and found there’s nothing here —regular doctors don’t deal with it. You must find a research team at a university, which is how I ended up at The University of Alabama at Birmingham. That was the only place I could get help (P 21)
Theme 5: Societal determinants of employment sustainability in long COVID
Subthemes	Illustrative Quotes
Public awareness and stigma	A lot of people just don’t know—and you don’t know what you don’t know. But it also feels like people just don’t care anymore (P 2)
	Some of them think you’re just being a hypochondriac—or that it’s all in your head (P 53)
	Especially I’ve had several doctors or specialists who have said, “Well, I can’t help you,” or “I don’t know anything about that and there’s no reason for me to see you again,” (P 45)
	In accommodation interview, she asked questions like how long is this condition going to last? It’s a chronic condition. It’s chronic. It’s pretty much permanent. There’s no cure for it (P 2)
Policy gaps	*Code: Stringent disability benefit eligibility and long waiting period*
	I won’t even qualify for short-term disability until October. I need help now, but I must work six months and a day just to get it (P 2)
	I was on short-term disability with my first employer. After that, I was denied long-term disability and SSDI due to lack of evidence. And the fact that I couldn’t work for 10 h a week is that evidence. I’m appealing both, but it’s been a long process (P 16)
	I’m still fighting for Social Security disability. Took two years to deny me. Now I have an appeal, which could take another year (P 58)
	*Code: FMLA [unpaid medical leave] exemption for small businesses*
	But unfortunately, working for a small non-for-profit business, they do not offer intermittent FMLA [unpaid medical leave] or long-term disability (P 45)
	*Code: Inconsistent state-level protections*
	We’re not forced unionism here in Arizona, so I didn’t really have any support (P 58)
Labor market challenging	During the pandemic, staff cuts were significant, and they never rehired. Many experienced workers were overlooked as companies hired for lower pay, including at my library. I even looked at jobs nationwide (P 58)
	I looked for a job for eight months after I quit. I still look. And there’s not anything available to meet my limitations now. I can’t drive, so it must be work at home. I’m going to have episodes, flare-ups, days where I can’t work, and what employer wants that (P 76)
	I applied to about 200 places over a year with no success. I thought maybe it was just the market (P 14)
Domain 3: Consequences of adverse employment outcomes in participants with long COVID	
Theme 6: The vicious cycle of employment, finances, and health	
Subthemes	Illustrative quotes
Financial strain	*Code: Job loss, income reduction, and rising healthcare needs drive financial instability*
	I went from zero credit card debt to now about $15,000 in credit card debt (P 58)
	I’ve had to use savings, credit cards, and pull from retirement and my kids’ college fund to get by (P 2)
	My pay hasn’t changed, but medical costs, including ER visits, specialist copays, and mental health appointments, have been a huge financial strain. Despite no hospitalizations, these bills have totaled thousands over the past three years (P 45)
	*Code: Informal support from family or friends*
	They occasionally give me Instacart or Walmart gift cards to help with food, but that’s all they can offer, as they have their own families and limited incomes (P 58)
	And if it had not been for the generosity of my mother-in-law, we’d have been selling the house, and moving, and doing a lot of things we didn’t want to (P 76)
	*Code: Bureaucratic barriers to accessing public assistance*
	I don’t qualify for food or government assistance because I still own my car, and I’m still fighting for Social Security disability, which took two years to deny me (P 58)
	Those with Long COVID and disability, it’s a real fight, and very few get accepted because there’s no test, there’s no medically diagnosed proof of Long COVID (P 45)
	*Code: Misaligned benefits and living costs*
	Even with SSDI at $18 K a year, that’s barely above the $15 K poverty line. To just stay afloat in L.A.—no extras, no vacations—you need about $90 K (P 16)
	*Code: Benefit system trade-offs and tax implications*
	I was approved for Social Security disability in November, but it reduces my long-term disability benefits, and the Social Security benefit is taxable, lowering my income (P 76)
Deterioration of health and identity	*Code: The impact of financial stress on health and identity*
	I was having seizures again because of the stress, of the financial stress (P 2)
	I feel guilty not contributing financially. I want to work again, but I’m not sure it’s possible (P 23)
	*Code: Cost barriers and limited insurance coverage restrict access to essential healthcare*
	I’m on a low dose of naltrexone, but I can’t afford it after the next three months (P 58)
	I no longer have regular insurance and am on Medicaid. My diabetes is out of control, and it took a year to get the appropriate medication approved. I can’t drive, so I rely on Medicaid transport, but drivers often cancel without notice. I can’t afford Uber (P 58)

*FMLA* = family and medical leave act [unpaid medical leave], *SSDI* = social security disability insurance

## Data Availability

No datasets were generated or analyzed during the current study.
